# Radiography students wishing to work in the field of radiation therapy: A French experience

**DOI:** 10.1016/j.tipsro.2025.100313

**Published:** 2025-04-29

**Authors:** S. Boisbouvier, F. Hermant, A. Béasse

**Affiliations:** a Radiation Oncology Dept., Centre Léon Bérard, Lyon, France; b Institut de formation des manipulateurs d’électroradiologie médical, Poissy Saint Germain intercommunal hospital, Poissy, France; c Institut des formations paramédicales du GHT Ile de France Sud, Sud francilien hospital, Corbeil Essonnes, France

**Keywords:** Education, Initial training, Radiation therapy, Barriers

## Abstract

Radiation therapy (RT) is one of the main treatment modalities for cancer. In France, radiation therapists (RTTs) undergo a combined training program in radiography, nuclear medicine, and RT. In the context of rising cancer cases and greater utilisation of RT, RT departments face a workforce shortage. This study investigates the interest of final-year radiography students in working in RT and explores deterrents to their career choice.

A survey was conducted in January 2024 among French radiography students who had completed at least 3 weeks of RT clinical placement. The survey included closed and open-ended questions on career preferences and highlighted barriers to working in RT. Data analysis included absolute number, frequency of students wishing to work in RT according to the age range of the students. A Chi square test was used to assess the influence of the age range on the desire to work in RT field. A thematic analysis of free-text responses was also performed.

The results indicated a low interest in a career in RT among students, with the main reasons being psychological and educational barriers. Enhancing RT-specific education, improving clinical placements, addressing emotional resilience, and modernising training programmes are crucial to attract future RTTs and address workforce shortages in this critical field.

## Highlights

• Only few radiography students were interested in the field of radiation therapy in France.
• Various reasons have been identified to explain this lack of interest including psychological and educational barriers.
• Enhancing RT-specific education, improving clinical placements, addressing emotional resilience, and modernising training programmes are crucial to attract future RTTs.

## Introduction

More than 50 % of cancer patients require Radiation Therapy (RT) as part of their treatment [1]. RT relies on an interdisciplinary team including radiation oncologists, medical physicists and radiation therapists (RTT) [2]. RTTs are responsible for the safe and accurate delivery of the radiation dose prescribed and of the clinical care and support of the patient throughout the treatment preparation, treatment delivery and immediate post treatment phases [3]. They also contribute to patient support, prevention and toxicity management. They can refer the patient to supportive care if required during the RT pathway. As a multidimensional modality, RT requires both technical expertise and a strong commitment to patient care.

In France, RTTs have an education and training programme that is combined covering diagnostic, imaging, nuclear medicine, electrophysiology and RT [4,5]. The teaching of RT spans the three years of training. The curriculum includes four Teaching Units (TU) dedicated to RT, totalling approximately 200 h. Two so-called integrative TUs foster the development of clinical reasoning in professional situations. In addition, there are numerous fundamental TUs including fundamental physics, oncology, and biology, and transversal TUs including hygiene, communication, ethics, and introduction to research. Only 6 weeks of clinical placement are mandatory in RT over the three years. Courses are organized by healthcare managers—sometimes with experience in RT—and are delivered either by RT professionals or by a manager with RT experience. Once graduating from one of the 49 colleges in France, professionals can work in one or multiple fields [6].

A healthcare professional shortage has been observed for a few years in all of these fields [7,8]. In the context of a paucity of health professionals, those qualified in a specific field may not easily change to another and attracting students into the field would address the shortage in RT.

The hypothesis of this study was that using modern equipment, the possibility of various tasks that RTT can have in RT (CT scan, treatment room, treatment planning), in brachytherapy, patient contact and working as part of a multidisciplinary team would make RT an attractive option.

Therefore, this work aimed to evaluate the interest of final year students in working in the field of RT in France. In order to consider possible solutions for encouraging students to work in RT, reasons that could discourage students from working in this field were identified.

## Materials and methods

### Survey design

This study utilised survey-based methodology. A 10-question survey was designed in French by two radiography students supervised by one RTT. It was divided into four parts:

– Section 1 focused on participant demographics consisting of one question regarding the age of the respondent,
– Section 2 focused on the clinical placement completed by the respondent. To be eligible to complete this questionnaire, respondents were required to have completed at least three weeks in clinical placement;
– Section 3 focused on future career plans and wish to work in RT consisting of the following yes/no question: “do you wish to work in the RT field after graduating?”.
– Section 4 explored the barrier to working in RT.

In this forth section, seven yes/no questions and one open question were included regarding reasons of discouragement to work in RT, based on aspects identified based on literature. The diversity in terms of patients who need RT can expose RTT and students to psychological difficulties [9,10]. RT practice can be also described as labour intensive with the technological complexity and the requirement to remain accurate and safe [10]. For students, these aspects might be psychologically difficult and it was evaluated with one question in this questionnaire. Moreover, physical difficulties can also be a reason. Patients may need help to set up, and dedicated material can be heavy to carry (i.e. immobilisation devices, phantom, patient bed, …) incurring some musculoskeletal disorders [11]. Physical difficulties were proposed as a reason that discourage students from working in RT in one question. In addition, working conditions and salary can also be a reason for not being willing to work in a field that were assessed with two other questions. Working conditions can be different between the fields of practice. In RT, the work is planned only during the week and sometimes on Saturday but it is crucial to ensure that all the fractions of RT sessions are completed in case of machine breakdown or maintenance. Furthermore, there is no clear evidence of any difference in terms of salary across the fields of practice with some contradictions between the reports and the changes can be occurred in a context of recruitment difficulties despite similar education [7]. On the other hand, as being responsible for the treatment deliverance, students might have a feeling to have a greater responsibility in RT than in another field. This aspect was proposed as another reason that could discourage students from working in RT and assessed in another question. Education and training in the context of combined program with only 6 weeks of clinical placement were also identified as a reason that could deter students from working in RT and were assess with two questions [12].

Finally, they could share other reasons in a final and open-ended question.

### Distribution of the survey

After obtaining approval by local institutional review board, the survey was created in Google forms. The survey was piloted by 2 students other than those who created it, and 1 RTT to enhance clarity and content coverage before it was released.

The target participants were all the final year students in 2024 in France who had already completed at least 3 weeks of clinical placement in an RT department, out of the minimum 6 weeks required in French training [5,13,14].

The survey was shared on social networks (Facebook and LinkedIn) by SB and sent to the “Comité d’Harmonisation des Centres de Formation de Manipulateur d’Electroradiologie Médicale “(CHCFMEM), a committee gathering all the French radiographer colleges, whose directors were invited to share the survey to the final year students. The survey was available between the 1st and the 31st of January 2024 with a reminder sent by email 7 days prior to the closing date.

### Sample size

Using the total population of final year students in 2024 estimated at 1350 by survey of the CHCFMEM, with a 95 % confidence level, and a 5 % margin of error, the total sample size of 300 was required to be representative of the target population [15].

### Data analysis

Data were collated using Microsoft® Excel® 2019 MSO (16.0.10411.20011) and statistical analysis was performed using R version 4.2.1 (2022-06-23). Quantitative data were presented as absolute numbers and frequencies. A Chi square test was used to evaluate the influence of age on the desire to work in the RT field. Statistical significance was set as (p < 0.05).

A thematic analysis as described by Braun et al. was conducted by the first author to analyse the anonymised responses of the open-ended question and identify emergent themes from them [16]. This method was used because, to our knowledge, there is no well-defined theory to explain the career choice of students and some reasons may not have been identified in the literature review carried out for the questionnaire. The process of data coding using a thematic approach involved:

– reading the free comments several times to be familiarised with them;
– labelling the chunks of text that had a meaning in relation to the desire or not to work in RT field, to generate initial codes and searching for themes that were relevant to this topic. This step of the process was refined throughout of the analysis of the “free comments”;
– reviewing, defining and naming themes and sub-themes [16].

Independently, a co-author also presented the anonymised “free comments” to an A.I (chat GPT version-4o) model to perform the same analysis and reinforce the process of coding carried out by the first author. A prompt was designed with the help of the GPT ‘prompt engineer’ by aitoolreport on the GPT-4o chatbot by Open AI.

The prompt commands a thematic analysis of the comments in three steps:

– Identify key concepts,
– Categorize the concepts and.
– Present the results in a table with the following columns: Category, Sub-category, Highlighted Words/Phrases.

The use of AI contributed to research triangulation with A.I considered as an additional member of the analysis team [17]. The two authors discussed both analyses to obtain the final version of the themes and sub-themes that are presented in the results part.

## Results

### Demographic data

Between the 1st and the 31st of January 2024, 309 responses were collected (response rate of 22.9 % as there are 1350 final year students in the French radiography colleges). As 6 respondents did not perform at least a 3-week of clinical placement when they answered the questionnaire, only 303 responses were eligible for analysis. 277 respondents were aged between 18 and 25 and 26 were 26 years old or more.

### Desire to work in RT field and reasons of declining to work in the RT field

Among the 303 responses studied, 61 respondents (20.1 %) wished to work in the RT field including 10 (38.5 %) who were more than 26 years old and 51 (18.4 %) were aged between 18 and 26 (Table 1). On Chi square analysis, those who were older than 26 years were more likely to wish to work in the field of RT than their younger counterparts (p < 0.05). Among the 242 respondents who did not wish to work in the RT field, 241 respondents filled out the forth section of the questionnaire. The reasons for their choice in descending order, were psychological difficulties (n = 110–45.5 %), practical education (n = 96–40.1 %), responsibility (n = 83–29.8 %), working conditions (n = 61–25.2 %), physical fatigue (n = 45–18.6 %) and salary (n = 45–18.6 %) (Table 1). On Chi square analysis, psychological difficulties were correlated to the age of the respondent and represented a significant reason for the respondents who were between 18 and 26 years old. 24 respondents (9.9 %) did not select any reason. 78 respondents (32.2 %) would have preferred practical education longer than 6 weeks in RT.

**Table 1:** Absolute number of the students who wish to work in the RT field and reasons given by the respondents who did not wish to work in RT.

Age range of the students	18–26 years	>26 years	Total	Chi square value	P-value
Absolute number of the students who wish to work in the RT field	51	10	61(20.1 %)	5.94	<0.05
Absolute number of the students who did not wish to work in the RT field	226	16	241(79.9 %)		
Reasons:					
• Psychological difficulties • Physical fatigue • Working conditions • Salary • Responsibility • Practical education	107 43 58 43 78 88	3 2 3 2 5 8	110 45 61 45 83 96	4.24 0.30 0.24 0.30 0.01 1.22	<0.05 0.58 0.63 0.58 0.92 0.27

### Additional reasons given

Among the 303 respondents, 99 wrote in the ‘free comments’ field. Based on thematic analysis, the themes and sub-themes identified in the “free comments” are presented in Table 2.

**Table 2:** Themes and sub-themes of the free comment analysis with relative number of phrases by theme.

Theme	Sub-theme
Specialisation Constraints (n = 17–13 %)	Early specialisation limits future options
	Loss of diagnostic skills and knowledge
	Limited technical procedures
	Lack of care procedures
Working conditions and environment (n = 56–42 %)	Job monotony
	Working conditions
	Lack of variety
	Atmosphere and team dynamics
Emotional and Mental Strain (n = 27–20 %)	High responsibility early on
	Mental burden and emotional impact of patient conditions
Educational Experience (n = 13–10 %)	Insufficient training
	Poor mentorship in clinical placement
Personal Preferences (n = 21–15 %)	Preference for diagnostic over therapy
	Disinterest in RT

## Theme 1: Specialisation constraints

This theme described the student’s perception regarding the specificity of RT in comparison to other fields of practice available to radiographers.

### Early specialisation limits future options

Some students described RT as a speciality that reduces the possibility to change to another field of practice especially at the beginning of their career.

“*we cannot practice in another field when we work in RT*” and RT, “*a speciality in its own right that ‘cuts us off’ from other specialities (medical imaging for instance)*”

### Loss of diagnostic skills and knowledge

They also mentioned a fear of losing skills such as “*anatomy*” and “*knowledge in the other fields of practice*” as well as a *“difficulty if I want to come back to diagnostic imaging”.*

### Limited technical procedures

Certain students described a lack of technical procedures with comments like *“not enough technical procedures”* and “*only few technical things”* in RT.

### Lack of care procedures

Several comments referred to a lack of care procedures in RT with students who mentioned a *“lack of care activities”* and a student who reported she preferred *“the care side of the job”.*

## Theme 2: Working conditions and environment

This theme included the student’s perception on the daily work in the RT team and gathered: Job monotony, working conditions, the atmosphere and team dynamics and the lack of variety were identified as sub-themes.

### Job monotony

Students reported the RTT practice as “*repetitive”*, “*monotone*” and even “*boring*”. RT was described as “*more repetitive than the diagnostic imaging*”.

### Working conditions

The working conditions were described as “*assembly line work*” “*without enough time with patient*”.

### Lack of variety

Moreover, students mentioned “*to always do the same things*” in RT and “*not to find out new things*”.

### Atmosphere and team dynamics

Finally, in the free comments were noted: “*teams are often tough*” and students reported that work atmosphere discouraged them from working in RT.

## Theme 3: Emotional and mental strain

This category includes the students’ feedback on the responsibility and mental burden with the emotional impact of the patient condition.

### High responsibility early on

Students reported a “*big responsibility at the beginning of the career*” with a fear “*not to make errors*”.

### Mental burden and emotional impact of patient conditions

They also described the difficulties to “*see sick persons every day*”, with “*the mental burden to patient follow up*” and “*the attachment to patients*”.

## Theme 4: Educational experience

This category describes the students’ perception regarding their educational background in RT including their clinical placement.

### Insufficient training

Students reported insufficient training: with comments like: “*lectures in RT are not detailed enough*”, “*lack of basic knowledge*”, and “*technical knowledge is not strong enough*”.

### Poor mentorship in clinical placement

Additionally, students mentioned a poor mentorship in clinical placement with “*RTT, not pedagogue*” and “*the bad experience in clinical placement*”.

## Theme 5: Personal preferences

This category includes all the free comments regarding the preference of the respondents for the diagnostic field and the disinterest in RT.

### Preference for diagnostic over therapy

Some students reported to be “*more interested in diagnostic imaging*” and to “*prefer diagnostic imaging rather than RT*”.

### Disinterest in RT

Several of them clearly mentioned “*I do not like RT*”, “*I do not like this field of practice*”, RT “*does not interest me*”.

## Discussion

This study evaluated the interest of radiography students in France to working in the field of RT. Only 61 out of the 303 participants representing 20.1 % indicated they were interested in the field of RT, which is very low. It partially explains the professional shortage of RTTs in France. To our knowledge, it is the first study on this topic likely due to several countries have dedicated undergraduate programmes in RT or at least one final year specialised in RT [12,18].

### Psychological challenges and responsibilities

A perception that the field of RT was highly psychologically draining was a reason reported by students for not wanting to work in RT which is consistent with the literature [9]. Half of all participants mentioned psychological difficulties in RT and 20 free text comments referred to the mental burden of RT as well as the emotional impact of the patient condition. Our results showed that psychologically difficulties are significantly in the youngest students suggesting that personal and or past professional experience could help to support, advocate for and assist patients with various tumour sites, stage, symptoms and treatment intent and diverse co-morbidities, psychological and social needs to face treatment, fear of toxicities, anxiety, pain. Typically, close relationships are established between RTTs and their patients. However, these types of relationships require specific skills in particular soft skills and could generate emotional distress that students must be taught to deal with [[19], [20], [21], [22]]. The relationship with patient constitutes an important aspect of the RTT role, a potential type of responsibility [18]. Indeed, the results of this study support the hypothesis that responsibility is a reason for radiography students for not wanting to work in RT. By definition, RTT are responsible for the accurate delivery of radiotherapy to cancer patients as part of a multidisciplinary team [3]. Facing emotional distress and assuming responsibilities are challenges where the training plays a crucial role and the French curriculum can benefit of the multiple positive experiences reported in the literature on this topic [[23], [24], [25]]. In addition, the establishment of a trusting relationship with colleagues in the teams is also very important to help professionals in their role toward the patient [26,27].

### Work environment and perception of automation in the RTT work

Unfortunately, the participants of this study reported a negative working atmosphere. More broadly, working conditions was found as a key factor in disinterest, with more than one in 4 students reporting this reason. However, the working conditions highlighted in the free comments did not primarily relate to the scheduling and the potential changes due to the machine breakdowns. Instead students reported a perception of RT as a repetitive and intensive activity where RTTs are required to treat a high volume of patients each day. In the past decades, technological development and innovation have radically changed the environment of RT departments enabling a personalisation of patient treatment and care. These developments have improved efficiency of the radiotherapy workflow, quality and reproducibility of the tasks and patient information [28]. Thus, modern machines with new ring-like design instead of C-arm gantry are common in the RT facilities, image guidance for radiation delivery and the automation of the couch position are now standard procedures with respect to beam delivery, and the use of adaptive RT has transformed treatment delivery in RT. However, as these developments include a greater level of automation, this may be perceived as a “simple press button” task albeit with still high responsibility to treat patients with large doses. These changes can intimidate students in particular who may be without a sufficient theoretical background in RT to understand the context of automation in RT [18]. It is potentially more important during the weeks of clinical placement where students will see what could be his/her future job if he/she chose to work in a RT department. While automation might be leading to certain tasks being considered repetitive, it also presents an opportunity to enhance RT treatment and allow RTTs to dedicate more time to patient care [19].

### RTT education

RTT education includes a part of theorical training and a practical education with clinical placement which is crucial for applying theoretical knowledge into practice [29,30]. However, our results indicate that clinical placement is a key reason for students who did not want to work in RT and negative working atmosphere reported could explains their refusal to work in RT. Moreover, the 6-week clinical placement was described being insufficient for students whose experience was limited to observation, as they did not have the opportunity to actively participate in practice. However, students are placed in RT departments to learn and the clinical placement should be organized with time for both initial observations and subsequently for active practice. RTTs have to take part of these educational tasks with the responsibilities in progression of the students [6]. To allow the best student’s experience, effective communication between educators, clinical mentors and students is crucial with objectives, relevant knowledge and skills collaboratively defined [18,19,30]. In the French context, the connection between educators and clinical mentors depends on the educators and the clinical mentors. Moreover, the definition of the objectives is performed with a common portfolio to radiography, nuclear medicine and RT and can be difficult to use in the context of RT.

More broadly, free comments suggested that RT education in France is insufficient, particularly in theoretical content such as physics. Theoretical education therefore needs to be strengthened. Combined programs remain a standard in the majority of European countries but the extent of RT-specific content is very varied [12]. In France, all colleges must follow a specified program. Its latest version was published in 2012 and upgraded in 2020 without modification in the RT content. The results of this survey indicate that it should be reviewed and actualized to encompass the technological developments in RT and the need for additional skills for students [5,13,14,21,29]. In addition, each college should have an RTT as educator to teach but also to coordinate the courses given by other professionals relative to the RT content as stated in the IAEA Handbook TD58 [30]. Educational content should be evidence based and incorporate diverse learning methods such as blended learning, virtual reality, and simulation to help students to acquire new skills and knowledge [[31], [32], [33], [34]].

### Is RT a specialisation?

Personnel preference was highlighted in the survey’s free comments as a factor influencing student disinterest in RT. A preference for diagnostic imaging may be directly or indirectly linked to previously discussed reasons while the connection between the perception of RT as a specialization and the previous reasons is subtler. Combined programs may give the advantage to allow graduates to start in one field and transition to another. However, students expressed concerns that choosing RT would limit their ability to switch fields of practice later in their careers. These insights reinforce the need to enhance RT education and promote the profession, supporting the idea of dedicated undergraduate RT programs or a final-year RT specialization [12,18].

### Minor reasons on the career choice

Finally, physical difficulties and salary were only minor factor reported in students uninterested in RT suggesting little to no difference between the future fields of practice. Regarding physical difficulties, Fernandes et al. found no significant variation in musculoskeletal disorder symptoms between radiographers and RTTs, further indicating that physical challenges are not unique to RT [11].

### Limitations

There are limitations in our study that need to be outlined. Gender of participants was not collected in the survey while it has been reported that male student could be less interested in working in RT than females [35]. Moreover, some respondents had only 3 weeks in clinical placement in RT which could be insufficient for students to have an opinion on their career choice.

Finally, alternate methodology such as focus groups with students may have provided more insight on the reasons as to why students do not wish to work in RT [36]. However, this was not possible due to time constraints. Despite this, the opportunity to add and analyse free comments in the survey reinforced the results of the closed-ended questions [37]. This work constitutes a first step in the exploration of this topic in France and focus groups will provide a future opportunity to work on a strategy to attract final-year students in RT.

At the end of this study, some questions remain unanswered. First, is the French model of three years sufficient for education in three specialities-radiography, nuclear medicine and RT? Second, do students have sufficient time to acquire all knowledge and skills necessary to safely practice in any of these fields of practice? [38]. This requires additional research.

## Conclusion

About only 20 % of final-year students are interested in working in RT in France contributing to the ongoing shortage experienced by RT departments. The main reasons reported are the psychological difficulties, the repetitive nature of RT tasks, the education, a preference for diagnostic imaging and the perception of RT as a specialization. RTTs, educators, radiation oncologists and medical physicists should work together to attract future RTTs and address workforce shortages in this critical field. This requires improvements in education such as enhancing soft skills training, clearly defining clinical placement objectives and monitoring the student’s progression, modernising training programmes to incorporate the latest advancements.

## Declaration of competing interest

The authors declare that they have no known competing financial interests or personal relationships that could have appeared to influence the work reported in this paper.
